# Advanced Neonatal Medicine in China: A National Baseline Database

**DOI:** 10.1371/journal.pone.0169970

**Published:** 2017-01-18

**Authors:** Xiang-Peng Liao, Selma Chipenda-Dansokho, Antoine Lewin, Nadia Abdelouahab, Shu-Qin Wei

**Affiliations:** 1 Centre Hospitalier Universitaire de Sherbrooke (CHUS) Research Centre, Sherbrooke, Quebec, Canada; 2 Department of Newborn, Wuxi Maternity and Child Health Hospital, Nanjing Medical University, Jiangsu, China; 3 Office of Education and Continuing Professional Development, Faculty of Medicine, Laval University, Quebec City, Quebec, Canada; 4 Department of Obstetrics and Gynecology, Saint-Justine Hospital, University of Montreal, Montreal, Quebec, Canada; Centre Hospitalier Universitaire Vaudois, FRANCE

## Abstract

Previous surveys of neonatal medicine in China have not collected comprehensive information on workforce, investment, health care practice, and disease expenditure. The goal of the present study was to develop a national database of neonatal care units and compare present outcomes data in conjunction with health care practices and costs. We summarized the above components by extracting data from the databases of the national key clinical subspecialty proposals issued by national health authority in China, as well as publicly accessible databases. Sixty-one newborn clinical units from provincial or ministerial hospitals at the highest level within local areas in mainland China, were included for the study. Data were gathered for three consecutive years (2008–2010) in 28 of 31 provincial districts in mainland China. Of the 61 newborn units in 2010, there were 4,948 beds (median = 62 [IQR 43–110]), 1,369 physicians (median = 22 [IQR 15–29]), 3,443 nurses (median = 52 [IQR 33–81]), and 170,159 inpatient discharges (median = 2,612 [IQR 1,436–3,804]). During 2008–2010, the median yearly investment for a single newborn unit was US$344,700 (IQR 166,100–585,800), median length of hospital stay for overall inpatient newborns 9.5 (IQR 8.2–10.8) days, median inpatient antimicrobial drug use rate 68.7% (IQR 49.8–87.0), and median nosocomial infection rate 3.2% (IQR1.7–5.4). For the common newborn diseases of pneumonia, sepsis, respiratory distress syndrome, and very low birth weight (<1,500 grams) infants, their lengths of hospital stay, daily costs, hospital costs, ratios of hospital cost to per-capita disposable income, and ratios of hospital cost to per-capita health expenditure, were all significantly different across regions (North China, Northeast China, East China, South Central China, Southwest China, and Northwest China). The survival rate of extremely low birth weight (ELBW) infants (Birth weight <1,000 grams) was 76.0% during 2008–2010 in the five hospitals where each unit had more than 20 admissions of ELBW infants in 2010; and the median hospital cost for a single hospital stay in ELBW infants was US$8,613 (IQR 8,153–9,216), which was 3.0 times (IQR 2.0–3.2) the average per-capita disposable income, or 63 times (IQR 40.3–72.1) the average per-capita health expenditure of local urban residents in 2011. Our national database provides baseline data on the status of advanced neonatal medicine in China, gathering valuable information for quality improvement, decision making, longitudinal studies and horizontal comparisons.

## Introduction

In 2015, there were about 1.4 billion people in China, accounting for about a fifth of the world population[1]. According to the 2010 census, there was about 15.9 million live births in China, with a crude birth rate of 11.9 births per 1,000 total population[2]. The report from the United Nations inter-agency group for child mortality estimation shows that neonatal mortality rates in China were 30 deaths per 1,000 live births in 1990, and 6 deaths per 1,000 live births in 2015[3]. Furthermore, data show that an almost 80% decline in premature mortality in children between 1990 and 2010[4], a 62% decrease in neonatal mortality between 1996 and 2008, and an improved international neonatal mortality standing. However, there were large discrepancies in neonatal mortality rates between urban and rural counties, and the three main diseases of birth asphyxia, preterm birth complications, and congenital abnormalities accounted for 64% of deaths in neonates in China during 2008[5–7].

It is well established that the decrease in neonatal mortality is explained by a number of intersecting factors such as politics, economics, culture and health beliefs, training and medical practice; nonetheless, advances in neonatal medicine are likely key elements of this trend. National surveys of neonatal medicine are therefore needed to inform neonatal health care policy and practice. National surveys of this kind have been conducted such as in America[8], and Canada[9]. In China, two national surveys on the situation of hospitalized neonates[10] and on the neonatal intensive care units (NICU)[11], have contributed to the development of guidelines on the structure and organization of neonatal wards[12]. There are also a number of reviews and retrospective studies of neonatal medicine[13, 14], and surveys on the morbidity and mortality of neonatal respiratory failure at the Chinese national level[15, 16]. However, there is still a need for more comprehensive studies that gather data on issues such as staffing, investment, health care practice, and health care costs, which are key to health policy decision-making.

Given China’s population, the country’s progress in neonatal medicine, the data and insight gathered from this experience can inform efforts to improve child health worldwide[17]. Comprehensive data on neonatal medicine in China will also help to explain the important decrease in child mortality in China in recent years[18, 19].

The present study seeks to answer two main questions: (1) from an international point of view, what detailed evidences or situations in China’s experience in the practice of neonatal medicine explain the speedy decline of neonatal mortality? (2) from a national perspective, what are the situations or differences in neonatal medicine across regions and hospitals? For these purposes, we established a national dataset of neonatal medicine in China, by integrating comprehensive data on tertiary neonatal medicine in China over three consecutive years (2008, 2009 and 2010), including investment in neonatal health, workforce, health activities, and disease expenditure. This database may serve as a benchmark for future longitudinal and cross-sectional studies.

## Methods

### Data sources

We extracted data from documents submitted as part of a request for proposals issued in 2011 by The Ministry of Health of the People's Republic of China (MOH) for projects aiming to evaluate and develop national key clinical subspecialty indicators in mainland China. And the indicators included the data for three consecutive years (2008–2010). All applicants were required to provide information on their institutional infrastructure, workforce, health-care, research, and training activities. Given the availability of electronic medical record systems at Class A level III hospitals in China, the highest level serving as medical hubs and being responsible for providing specialist health services, medical education and research, database administrators of the hospitals were able to export objective data from hospital information system[20].

**Fig 1:**
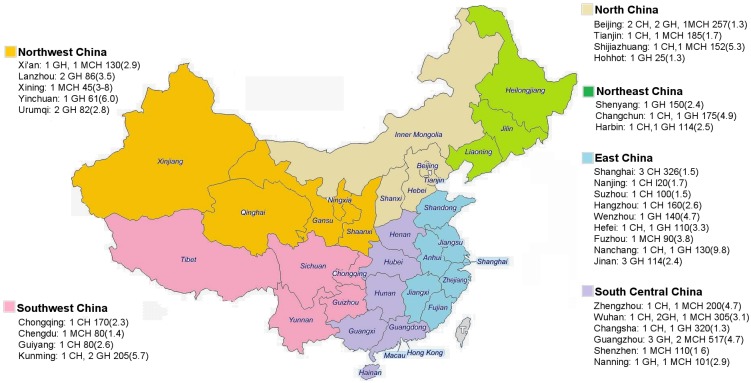
Distribution of 61 surveyed newborn units in China. Sixty-one surveyed newborn units were from Class A level III provincial or ministerial affiliated hospitals, distributed in 31 cities of 28 provincial districts among six geographical regions in mainland China. The units were from child hospital (CH), general hospital (GH), and maternal and child hospital (MCH). The city names, hospital types, newborn beds, and newborn bed-population ratios (number in the brackets, for every 100,000 population) were marked respectively. The adapted map is for illustrative purposes only, originally from the free maps website (http://www.d-maps.com/carte.php?num_car=11572&lang=en).

Of the 31 provincial districts in mainland China, three provinces (Shanxi, Hainan, and Tibet), accounting for 3.6% of population of mainland China and that were relatively underdeveloped with respect to socioeconomic and health[21], were absent from this application. In all, 61 newborn units in Class A level III provincial or ministerial hospitals (see Fig 1 and S1 Table), affiliated with universities with the most advanced level in medicine, education and research within local areas, were included. The newborn units were located in 31 cities (including one national capital, and 27 provincial capitals) within 28 provincial districts, and represented key provincial clinical institutions of neonatal medicine. Given these characteristics, findings from the study reflect the performance of advanced neonatal medicine in mainland China.

### Procedures

Proposals submitted in response to the request for proposals, and commitments of data authenticity were included in the study. Accepted proposals were then reviewed by representatives of provincial departments of health. All proposals accepted at this first stage of review were then forwarded to the Chinese Medical Association for further review and selection. Announced and posted by Chinese Medical Association on its official website, proposals were also opened to professionals and the public for data authenticity.

The study was not a random sampling survey, and we retrospectively extracted data from the 61newborn units in 2014 and 2015. To ensure data quality, related references such as the database of China National Knowledge Infrastructure, National Natural Science Foundation of China, Science Citation Index, MEDLINE/PubMed resources, and hospital websites, were searched. Furthermore, the corresponding author had access to individual hospital information during and after data collection.

### Indicators

We examined tertiary neonatal data for the years 2008, 2009, and 2010, including newborn health infrastructure, workforce, health service, and disease expenditure.

Data on newborn health infrastructure and workforce were the situation at the end of 2010. According to the National Bureau of Statistics of China, all provinces and municipalities are classified into six geographical regions (North China, Northeast China, East China, South Central China, Southwest China, and Northwest China). In China, the majority of newborn medical services are offered by children’s hospitals (CH), general hospitals (GH), and maternal and child hospitals (MCH), and the data were collected by year, type of hospital, and city or region.

Available newborn beds were the number of beds registered at health authorities. Investments on newborn units primarily represented the money for the purchase of medical equipment, scientific research, personnel training, and newborn ward reconstruction, and excluded the salary of staff. Health workforce included full-time professionals with certificates issued by The Ministry of Health of China, and each was just able to practice at a single hospital, having no authority to practice in other hospital wards. The workforce did not include people in training such as interns, trainee nurses, fellows, and ward care workers. Health professionals consisted of physicians (including chief physicians, deputy chief physicians, attending physicians, and resident physicians), and nurses (including chief nurses, associate chief nurses, nurse-in-charges, and primary nurses). Given that physician assistants, respiratory therapists, physiotherapists, pharmacists, and nutritionists are rare in newborn units in China, we excluded them for this investigation. The physician-to-bed ratio, nurse-to-bed ratio, and ratio of nurses to physicians were calculated in this study.

Diseases were classified according to the International Classification of Diseases, 9th Revision (ICD-9). Discharged patients were the number of sick newborns’ admission registrations at Hospital Inpatient Management System, and all deceased inpatients were counted as discharges. Length of hospital stay was defined as the number of nights the sick newborn remained in the hospital for his or her stay, and length of hospital stay for a particular disease in a hospital was calculated by dividing the sum of inpatient days by the number of patient admissions with the same diagnosis classification[22]. The number of patients who died in hospital, was small compared to overall discharges, and they were excluded for the calculation of length of stay. The definition of nosocomial infection was the same as that used by the Centers for Disease Control and Prevention[23].

Because disease spectrums were different across hospitals, and the numbers of discharged extremely low birth weight (ELBW) infants (Birth weight <1,000 grams) were missing for some hospitals, we chose typical parameters and diseases for the analysis of disease expenditure. For survival rate of ELBW infants, we calculated the percentage from five hospitals in 2010, where each unit had more than 20 discharged ELBW infants reported yearly. Based on clinical system, X-ray and laboratory examination, the diagnostic criteria of respiratory distress syndrome (RDS), pneumonia, and sepsis, were according to Chinese textbook of Practice of Neonatology (Third edition)[24], and they were similar to the ones in The Merck Manuals[25–27].

Disease specific medical expenditure was estimated as the cost of principal discharge diagnosis for a hospital stay, regardless of other diagnoses, charged to a hospital finance department for newborn care, not including the cost for parents’ inpatient care. The value of the Chinese Yuan (CNY) (Renminbi) was adjusted to United States Dollars (USD) using the consumer price index (1 USD = 6.623 CNY on December 30, 2010).

The ratio of hospital cost to per capita disposable income of urban residents was calculated by dividing disease-specific medical expenditure by per-capita disposable income of local urban residents. The ratio of hospital cost to per-capita health expenditure of urban residents was calculated by dividing disease-specific medical expenditure by local per-capita health expenditure. The data on disposable income of local urban residents, and per-capita health expenditure of local urban residents in China were obtained from the China Statistical Yearbook 2013[28].

### Analysis

First we conducted descriptive statistics to explore the distribution and frequencies of results. Given the non-normal distributions of variables, the descriptive parameters reported are the median, interquartile range (IQR), range (minimum and maximum), and sum if necessary. Then we conducted Independent-Samples Nonparametric Test for variables with non-normal distribution. Post hoc analysis, such as pairwise comparison, was omitted, because our main purpose was to establish a national baseline database. Data were analyzed using The Statistical Package for Social Sciences (SPSS, version 23.0).

## Results

### Available newborn beds

Of the 61 newborn units in Class A level III provincial or ministerial hospitals, 20 were CHs, 29 were GHs, and 12 were MCHs. The newborn units were located in 31 cities, six regions within China (Fig 1). There was a wide range (20–260 beds) in the number of newborn beds across the hospitals (median = 62 [IQR 43–110]) at the end of 2010. South Central China had the highest proportion of newborn beds (31%), followed by East China (28%), North China (13%), Southwest China (11%), Northeast China (9%), and Northwest China (8%). The proportion of newborn beds in CH, GH and MCH were 44%, 33% and 24% respectively (Table 1).

**Table 1:** Newborn unit investment, workforces and health care activities in 61 hospitals during 2008–2010.

	Median	IQR	Range	Sum
Available newborn beds in 2010	62	43–110	20–260	4,948
Child hospital (*n* = 20)	100	73–143	40–200	2,167
General hospital (*n* = 29)	50	37–60	20–150	1,617
Maternal & Child hospital (*n* = 12)	85	64–114	32–260	1,164
Investment (1000 US$)	345	166–586	11–3,510	83,622
2008	302	151–508	29–3,510	27,995
2009	318	170–586	11–2,225	26,137
2010	364	180–695	18–1,812	29,490
Number of physicians with degree in 2010	22	15–29	8–44	1,369
PhD	3	1–8	0–20	279
Master Degree	9	6–15	0–29	686
Bachelor Degree	5	2–10	0–23	394
Number of nurses with degree in 2010	52	33–81	12–126	3,443
PhD	0	0–0	0–1	2
Master Degree	0	0–1	0–5	40
Bachelor Degree	16	10–27	3–74	1,272
College	31	18–45	1–101	2,129
Discharged inpatients	2,284	1,266–3,522	438–7,530	465,629
2008	2,158	1,169–3,301	438–5,434	142,434
2009	2,327	1,234–3,659	532–6,409	153,036
2010	2,612	1,436–3,804	616–7,530	170,159
Length of hospital stay for overall newborns (Days)	9.5	8.2–10.8	4.2–17.5	
2008	9.7	8.3–11.4	4.2–14.8	
2009	9.5	8.2–10.5	5.2–16.8	
2010	9.3	7.9–10.6	4.9–17.5	
Inpatient antimicrobial drug use rate (%)	68.7	49.8–87.0	11.5–100	
2008	70.5	52.8–88.5	15.2–100	
2009	69.9	53.9–85.8	14.5–100	
2010	65.0	44.4–85.6	11.5–100	
Nosocomial infection rate (%)	3.2	1.7–5.4	0.0–14.9	
2008	3.2	2.0–5.5	0.1–14.9	
2009	3.1	1.7–5.4	0.0–12.0	
2010	3.1	1.6–5.3	0.0–12.1	

IQR, Interquartile range; Range (minimum-maximum)

### Investments on newborn units

During 2008–2010, there was an overall US$83.6 million investment in the newborn units. The median yearly investment for a single newborn unit was US$344,700 (IQR166,100–585,800). MCHs invested more than CHs and GHs (*P* = 0.016), and the median yearly investments for MCH, CH and GH were US$475,200 (IQR 181,200–742,100), US$413,000 (IQR 236,200–659,200), and US$245,600 (IQR 151,000–456,100) respectively.

### Newborn health workforce

The median number of physicians per newborn unit was 22 (IQR 15–29), with a total of 1,369 across the 61 hospitals. There were more physicians in CHs (median = 26, IQR 22–35) (*P* = 0.027), followed by MCHs (median = 22, IQR17-33) and GHs (median = 15, IQR 12–25). The proportions of chief physicians, deputy chief physicians, attending physicians, and resident physicians were 19.4% (265), 19.4% (266), 28.9% (396) and 32.3% (442), respectively. There were significant differences in job titles across regions (*P* = 0.040) and hospital types (*P<*0.001).

Among all physicians, 20.4% (279) had M.D/PhD degrees. Physicians with the degree of Master of Medicine shared 50.1% (686). There were significant differences in academic attainment for physicians across hospital types (*P<*0.001), but not across regions. 30% (153) of physicians had M.D/PhD degrees in GH, while 15% (85) in CH and 15% (41) in MCH.

Our results showed that there were 3,443 nurses in the newborn units, and the nurse-to-bed ratio was 0.70 (1: 1.44). No significant differences in nurse-to-bed ratio were found across regions and hospital types. The median of nurses per unit was 52 (IQR 33–81). Only 2.6% (90) of nurses had senior job titles (chief nurse, and associate chief nurse), and 80.8% (2,782) of nurses were in junior positions. The majority (61.8%) of nurses were trained at the college level and only two nurses had a PhD degree, and 42 nurses had a Master’s degree. No significant differences in academic attainment for nurses were found across regions and hospital types.

Overall, the physician-to-nurse ratio was 0.4(1: 2.51), and the physician-to-bed ratio was 0.27 (1:3.61).

### Inpatient discharges and length of hospital stay

During 2008–2010, there was a total discharge of 465,629 newborn inpatients, and the overall inpatient discharges increased by 19.5%. In 2010, the median inpatient discharges in newborn units were 2,612 cases (IQR 1,436–3,804), and one physician annually discharged 117 (IQR 81–164) inpatients, and one nurse care about 48 (IQR 38–60) inpatients. The median length of hospital stay for overall inpatient newborns was 9.5 (IQR 8.2–10.8) days (see Table 1). And the median length of hospital stay depended on the type of condition: 14.04 (IQR 11.0–16.8) days for preterm infants (less than 37 weeks’ gestation); 22.6 (IQR 15.3–31.0) days for very low birth weight (VLBW) infants (Birth weight <1,500 grams) (Table 2).

**Table 2:** Disease expenditure of a single hospital stay for common newborn diseases during 2008–2010.

	Length of hospital stay (Days)				Daily cost (US Dollars)				Hospital cost (US Dollars)			
	Pneumonia	Sepsis	RDS	VLBW infants	Pneumonia	Sepsis	RDS	VLBW infants	Pneumonia	Sepsis	RDS	VLBW infants
North China	8.6	14.0	14.3	14.8	124	112	194	153	1,046	1,714	2,540	2323
	(7.3–10.3)	(10.5–15.6)	(10.1–25.6)	(8.4–21)	(117–155)	(104–184)	(141–272)	(124–206)	(871–1,423)	(1,045–2,577)	(1,541–4,989)	(1,028–4,440)
Northeast China	7.5	10.1	10.2	21.5	98	124	151	152	750	1,242	1,474	2,842
	(7–8.3)	(9.6–10.6)	(9–14)	(14.8–25.6)	(85–111)	(120–135)	(128–223)	(129–193)	(675–804)	(1,146–1,432)	(1,206–2,424)	(2,518–3,329)
East China	9.0	14.0	16.3	30.0	95	92	139	128	820	1,161	2,647	3,540
	(7.3–10.3)	(11.3–15.1)	(12.5–22.3)	(21–36.1)	(70–102)	(75–115)	(107–189)	(109–157)	(642–1,078)	(945–1,548)	(2,353–3,166)	(3,058–4,833)
South Central China	10.2	14.8	16.7	31.4	102	136	174	162	1,113	1,858	3,518	4,630
	(8.3–13.4)	(13.7–17.3)	(13.1–23.6)	(24.4–35.8)	(75–136)	(90–157)	(154–229)	(113–207)	(749–1,426)	(1,401–2,603)	(2,613–4,227)	(2,605–6,571)
Southwest China	9.3	10.4	11.6	15.3	99	118	171	134	819	1,046	2,461	2,023
	(7.4–10.9)	(9.5–15.1)	(8.5–16.1)	(13.1–18.1)	(79–116)	(86–128)	(120–293)	(102–154)	(697–1,043)	(890–1,684)	(1,275–2,862)	(1,405–2,579)
Northwest China	9.0	11.5	12.0	15.1	99	138	174	151	681	1,550	1,891	1,963
	(5–11.5)	(9.1–15.9)	(10–14.2)	(11.7–20.1)	(82–125)	(96–148)	(135–212)	(99–192)	(499–1,201)	(1,269–1,639)	(1,480–2,253)	(1,397–3,586)
Over all	9.0	14.0	14.3	22.6	99	109	164	141	856	1,369	2,575	3,306
	(7.4–10.8)	(10.9–15.3)	(11–20.0)	(15.3–31.0)	(84–119)	(88–136)	(128–222)	(114–185)	(682–1,162)	(1,044–1,767)	(1,619–3,516)	(2,048–4,629)
*P*-value	0.008	0.114	0.002	<0.001	0.006	0.005	0.137	0.393	<0.001	0.008	<0.001	<0.001

*P*-values were the probabilities compared across six regions within China; Interquartile ranges in the brackets were marked respectively. RDS, Respiratory distress syndrome; VLBW, Very low birth weight (Birth weight <1,500 grams).

### Nosocomial infection and use of antibiotics in hospitalized patients

During 2008–2010, nosocomial infection rates (median = 3.2% [IQR 1.7–5.4]) varied largely across the hospitals, and at one GH, the hospital infection rate peaked to 14.9% (see Table 1). No significant differences in nosocomial infection rate were found across hospital types and regions. However, the use of antibiotics was significant different across hospital types (*P* = 0.025) and regions (*P* = 0.008). Overall, about two-thirds (median = 68.7% [IQR 49.8–87.0]) of newborns received antibiotics during their stays in hospital, and the median in CHs (median = 81.0% [IQR 49.6–92.1]) was higher than GHs (median = 65.0% [IQR 51.9–76.9]) and MCHs (median = 67.9% [IQR 36.8–89.4]). Over the three years, the nosocomial infection remained same, and the use of antibiotics in hospitalized patients dropped by 5.5% (Table 1).

### Cutting-edge neonatal technologies and quality

Advanced incubators and ventilators, cardio-respiratory monitors, magnetic resonance imaging, and cardiac echocardiography, were commonly reported in most of the hospitals. Four hospitals in Hangzhou, Shanghai, and Guangzhou, used extracorporeal membrane oxygenation technology to care for the 17 sickest newborns during 2007–2010. Surgical repair of serious congenital cardiac malformations, complex thoracoabdominal surgery, minimally invasive surgery of gastrointestinal malformations, and hemodialysis were offered at several hospitals.

### Disease expenditure

**Fig 2:**
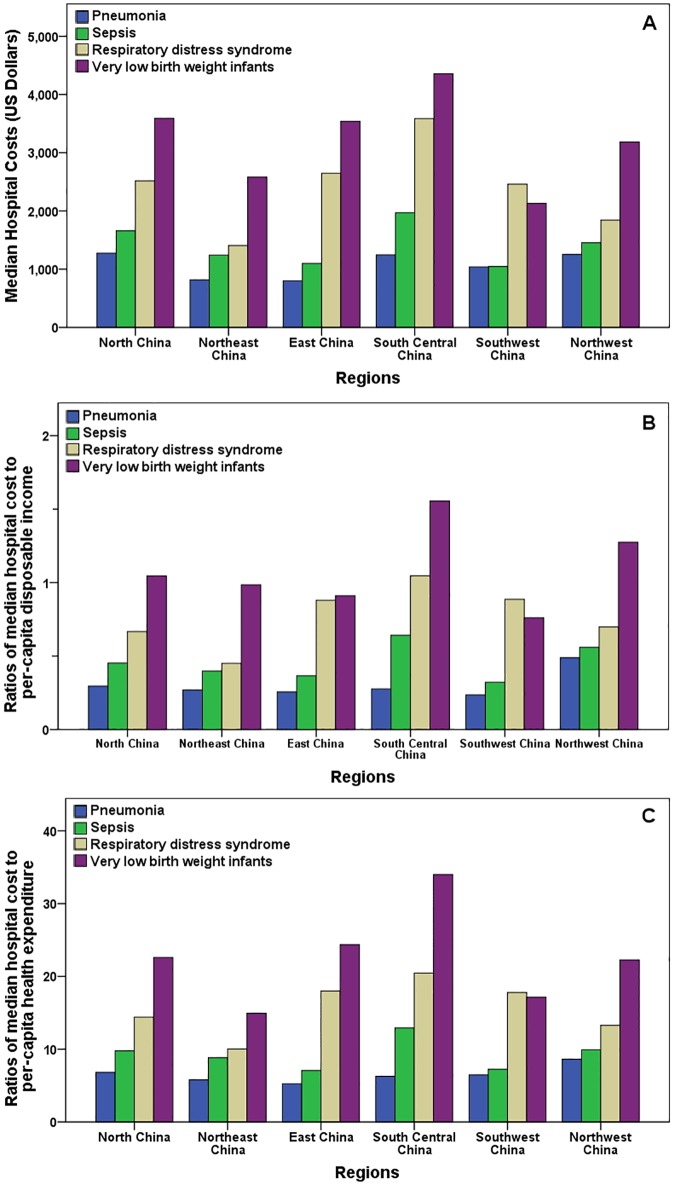
Disease expenditures of four common newborn diseases by regions during 2008–2010. (A) Median hospital costs by regions, and the *P*-values were all less than 0.001for regional comparisons of pneumonia, sepsis, RDS, and VLBW infants. (B) Ratios of median hospital cost to per-capita disposable income of local urban residents by regions, and the *P*-values for regional comparisons of pneumonia, sepsis, RDS, and VLBW infants were 0.237, 0.026, 0.003, and 0.092 respectively. (C) Ratios of median hospital cost to per-capita health expenditure of local urban residents by regions, and the *P*-values for regional comparisons of pneumonia, sepsis, RDS, and VLBW infants were 0.111, 0.001, less than 0.001, and less than 0.001 respectively.

During the three years, the length of hospital stay, daily cost, and hospital cost for common newborn diseases of pneumonia, sepsis, RDS, and VLBW infants, were specifically reported, and they all varied significantly across regions (Table 2), but not across hospital types. And for these diseases, the ratios of median hospital cost to per-capita disposable income of local urban residents, and ratios of median hospital cost to per-capita health expenditure of local urban residents, were significantly different across regions (Fig 2).

### Extremely low birth weight (ELBW) infants

During 2008–2010 in the five hospitals in four cities (Shenyang, Shanghai, Changsha, and Tianjin), the lightest infant who survived had a birth weight of 500 grams[29], but no infant with a gestation age of less than 25 weeks survived during this period. The median length of hospital stay, daily cost, and hospital cost for a single stay of hospital were 49.0 days (IQR 45.0–58.2), US$172 (IQR 148–196) and US$8,613 (IQR 8,153–9,216), respectively, and the survival rate of ELBW infants was 76.0% (259/341).

Our data also showed that the treatment of ELBW infants presented economic consequences to parents. Overall, the median hospital cost of ELBW infants for a single hospital stay was 3.0 times (IQR 2.0–3.2) the per-capita disposable income of local urban residents in 2011, or 62.9 times (IQR 40.3–72.1) times the per-capita health expenditure local urban residents in 2011.

## Discussion

This report summarizes the situation of the most advanced neonatal medicine in 28 of 31 provincial districts in mainland China, which accounts for 96% of the population of mainland China, with a discharge of 465,629 newborn inpatients in three consecutive years. We have collected comprehensive information through a national baseline database that includes indicators such as health investment, workforce, health care practice, and disease expenditure.

### Progress in neonatal medicine in China

China has made great progress in neonatal medicine in the last 30 years. Since the first NICU was established in Hangzhou, China in 1983, when there were only four NICU beds [30], neonatal medicine has been thriving in China[11, 13, 14]. Due to the availability of medical equipment such as ventilators, vital sign monitors, incubators, together with the progress in newborn nutrition, imaging and surgery, some level III hospitals in China can implement neonatal clinical technologies conducted in advanced countries[11, 31]. And cutting-edge neonatal technologies have been successfully operated in several hospitals with high success rates, such as extracorporeal membrane oxygenation[32]. Accompanied with clinical care, national neonatal education and training programs have been implemented, such as the China Neonatal Resuscitation Program conducted with the Johnson & Johnson Pediatric Institute in the USA[33], the Training Program in Neonatal-Perinatal Medicine in Shanghai with the Canadian Neonatal Network[34], and the Acute Care of At-Risk Newborns Program in Zhejiang with Canada[35]. Jointly, these advances have cumulatively led to the speedy decreases in neonatal, infant and under-5 mortalities in China[3–5, 7, 19].

### Comparisons of survival rates between countries

A big difference in China exists in the quality of tiny infant care compared with international advanced level. In addition to findings from present study that show 76% of survival rate in ELBW infants from five hospitals, reports from China further show that the survival rate of ELBW infants was 41%, and VLBW infants 82% in 109 hospitals in 2009[11], and that VLBW infants with neonatal respiratory failure was 58% in 55 NICUs during 2009–2010[15]. These data seem to be comparable with India, where has the similarity of large population size and the survival rate of ELBW infants (52%[36] and 56%[37] from two NICUs). However, no infants with a birth weight of less than 500 grams or gestational age of less than 25 weeks survived in China before 2011. Although a new neonatal medicine record was established in 2015 that an infant with a birth weight of 500 grams and gestational age of 24 weeks survived in Shenzhen, China[38], too many of the sickest newborns cannot receive successful treatments to discharge, with the result of abandonment[15]. In contrast, in the advanced international level as the USA, the survival of ELBW infants was approximately 90% during the entire 1990s[39], In Japan, the survival rate of birth weight of 750–999 grams was more than 90% in 2005[40]. Meanwhile, due to the varieties of gestational age at delivery and birth weight criteria for livebirth registration[41, 42], it is very much possible that the mean birth weight and gestation age of the ELBW infants from this data are larger than that from the reports in Japan[40], America[43], and Canada[44].

### Regional discrepancy in China

The data point to a clear regional discrepancy in neonatal healthcare outcomes (Fig 2). The report shows the huge differences in access to health services and financial protection across regions in China, such as in antenatal care coverage[45]. Reflecting the current socio-economic status of regions in China[21], most of the resources, such as high quantify-training professionals, advanced equipment and research funding, are concentrated in some metropolises, especially in the cities of Yangtze River Delta region, Pearl River Delta region, and Bohai Sea Ring Area. This also leads to obvious disproportion in treatment effects and hospital cost as found in this study.

Inadequate investment on health care in China has been long concerned. As found in this study, the total investment of less than 90 million dollars during the three years on the units with the discharge of 465,629 newborn inpatients, illustrates its serious shortage of funds. There are reports describing the serious working environment and shortage of pediatricians and nurses in China[46, 47]. Furthermore, there were no respiratory therapists and NICU social workers, and limited dietitians, pharmacists, and physiotherapists working in the newborn units in this study, in spite of the interdisciplinary nature of the needs of these infants. Another problem is that there is no insurance for newborn diseases in the majority of cities in China[48], and that may be one of main causes of withdrawal from treatment. One report about outcomes for very immature newborns in China finds that 46.2% of the deaths from withdrawal of support were associated with the parents’ inability to afford the high cost of continued medical treatment[15].

### Economic impacts of preterm birth

The expenditures for the treatment of preterm birth in China is much less compared to some high-income countries, but it is expensive compared to the medium Chinese family income. As reported, the length of hospital stay for VLBW infants was 53.7±38.4 days, and the cost was US$82,900±88,400 in California and New York during early1990s[49], but the length of hospital stay for VLBW infants in the USA is longer than that of ELBW infants (median = 49.0 days [IQR 45.0–58.2]) in this study, and the cost for VLBW infants in the USA is about ten times the amount of ELBW infants (median = US$8,613 [IQR 8,153–9,216]) in China. The shortage of neonatal health staffs in China, as well as the difference in service quality, partially contribute to this fact. But even so, the cost of ELBW infants in this dataset is three times the per-capita disposable income of local urban residents in China, or 62.9 times of per-capita health expenditure of local urban residents in China. In other words, it costs one and half years’ income of a pair of working parents, or 63 personals’ health expenditure to successfully discharge an ELBW infant from hospital to home for the first time. This does not include expenditures related to family stay at hospital, and the cost of other diseases resourced from preterm birth. Another report also shows the mean hospital cost of US$5,586 for preterm infants with gestational age of less than 28 weeks and neonatal respiratory failure during 2004 in China[16].

### Suggested policies for neonatal medicine in China

Every Newborn Action Plan issued by The World Health Organization, provides direction for ending preventable newborn deaths worldwide[50]. Given current situation in neonatal medicine in China, there are some priorities for the neonatal medical professionals to handle. One of urgent issues to deal with is the standardization and updating of disease management, especially in some critical diseases. In this study, variations in the length of hospital stay and disease expenditure, and the high rates of nosocomial infection and inpatient antimicrobial drug use, suggest inequalities in the provision of healthcare hospitals, cities and regions. More applicable recommendations, clinical guidelines, clinical paths and multidisciplinary cooperation are essential for the successful management and treatment of newborn diseases. In addition, it is important to strengthen continuing education at different levels, and to develop national and regional safety and quality assessment system. A successful example of disease management is The Neonatal Resuscitation Program in China, in which neonatal health care professionals received standard training in 20 target provinces, with 94% of delivery facilities and 99% of counties reached, and the intrapartum-related deaths in the delivery room decreased from 7.5 to 3.4 per 10,000 over a period of five years[33].

### Study limitations

There are some limitations in our study. Though the number of applicants by region and the regional ratios of newborn beds are complied with current population and socioeconomic status in China, there was no population-based sampling, and we relied on administrative data. Although the participating hospitals can be representatives of the highest level of neonatal medicine in China, a couple of hospitals with some strengths have missed this survey. Furthermore, while we summarize the status of advanced neonatal medicine, these results cannot be generalized to primary and secondary neonatal medicine in China. To minimize inaccuracies, we reviewed all the documents and searched well-established databases. We also collected another comprehensive dataset of research and training.

## Conclusions

In summary, this study provides a comprehensive baseline picture of neonatal medicine in China and describes a population-based database that can serve as a benchmark not only for further longitudinal and cross-sectional studies, but also provides important information to inform newborn practice and policy.

## Supporting Information

S1 Table List of participating hospitals. (DOCX)

## References

[1] Population Division, Department of Economic and Social Affairs, United Nations. World Population Prospects: The 2015 Revision, Key Findings and Advance Tables. New York: United Nations; 2015 19 p.

[2] National Bureau of Statistics of China. China 2010 population census data Beijing: National Bureau of Statistics of the People's Republic of China; 2012. http://www.stats.gov.cn/tjsj/pcsj/rkpc/6rp/indexch.htm.

[3] The United Nations Children's Fund. Levels and trends in child mortality. Report 2015. Estimates developed by the UN Inter-agency Group for Child Mortality Estimation. 2015 September 2015. p. 19.

[4] Yang G, Wang Y, Zeng Y, Gao GF, Liang X, Zhou M, et al Rapid health transition in China, 1990–2010: findings from the Global Burden of Disease Study 2010. Lancet. 2013;381(9882):1987–2015. Epub 2013/06/12. 10.1016/S0140-6736(13)61097-1 23746901 PMC7159289

[5] Rudan I, Chan KY, Zhang JS, Theodoratou E, Feng XL, Salomon JA, et al Causes of deaths in children younger than 5 years in China in 2008. Lancet. 2010;375(9720):1083–9. Epub 2010/03/30. 10.1016/S0140-6736(10)60060-8 20346815

[6] Wang H, Liddell CA, Coates MM, Mooney MD, Levitz CE, Schumacher AE, et al Global, regional, and national levels of neonatal, infant, and under-5 mortality during 1990–2013: a systematic analysis for the Global Burden of Disease Study 2013. Lancet. 2014;384(9947):957–79. Epub 2014/05/07. 10.1016/S0140-6736(14)60497-9 24797572 PMC4165626

[7] Feng XL, Guo S, Hipgrave D, Zhu J, Zhang L, Song L, et al China's facility-based birth strategy and neonatal mortality: a population-based epidemiological study. Lancet. 2011;378(9801):1493–500. Epub 2011/09/20. 10.1016/S0140-6736(11)61096-9 21924764

[8] Pollack LD, Ratner IM, Lund GC. United States neonatology practice survey: personnel, practice, hospital, and neonatal intensive care unit characteristics. Pediatrics. 1998;101(3 Pt 1):398–405. Epub 1998/03/14. 9481004 10.1542/peds.101.3.398

[9] Lee SK, McMillan DD, Ohlsson A, Pendray M, Synnes A, Whyte R, et al Variations in practice and outcomes in the Canadian NICU network: 1996–1997. Pediatrics. 2000;106(5):1070–9. Epub 2000/11/04. 11061777 10.1542/peds.106.5.1070

[10] Wei K-L, Yang Y-J, Yao Y-J, Du L-Z, Wang Q-H, Wang R-H, et al Epidemiologic survey on hospitalized neonates in China. Translational Pediatrics. 2012;1(1):15–22. 10.3978/j.issn.2224-4336.2011.10.01 26835259 PMC4728849

[11] Feng ZC, Coordination Group for Present Situation of Neonatal Subspecialty in the Mainland of China. Present situation of neonatal subspecialty in the mainland of China: a survey based on 109 hospitals. Chinese Journal of Pediatrics. 2012;50(5):326–30. Epub 2012/08/14. 22883031

[12] Feng ZC. Classification, construction and management of neonatal wards in China (Recommendation). Chinese Journal of Applied Clinical Pediatrics. 2013;28(003):238–40.

[13] Sun B, Shao X, Cao Y, Xia S, Yue H. Neonatal-perinatal medicine in a transitional period in China. Archives of disease in childhood Fetal and neonatal edition. 2013;98(5):F440–4. Epub 2013/06/14. 10.1136/archdischild-2012-302524 23759518 PMC3756438

[14] Du LZ, Xue XD, Chen C. Development course of neonatal medicine in China. Chinese Journal of Pediatrics. 2015;53(5):321–3. Epub 323. 26080658

[15] Wang H, Gao X, Liu C, Yan C, Lin X, Yang C, et al Morbidity and mortality of neonatal respiratory failure in China: surfactant treatment in very immature infants. Pediatrics. 2012;129(3):e731–40. Epub 2012/02/15. 10.1542/peds.2011-0725 22331337

[16] Qian L, Liu C, Zhuang W, Guo Y, Yu J, Chen H, et al Neonatal respiratory failure: a 12-month clinical epidemiologic study from 2004 to 2005 in China. Pediatrics. 2008;121(5):e1115–24. Epub 2008/05/03. 10.1542/peds.2006-2426 18450855

[17] Liu L, Oza S, Hogan D, Perin J, Rudan I, Lawn JE, et al Global, regional, and national causes of child mortality in 2000–13, with projections to inform post-2015 priorities: an updated systematic analysis. Lancet. 2015;385(9966):430–40. Epub 2014/10/05. 10.1016/S0140-6736(14)61698-6 25280870

[18] Mulholland K, Temple B. Causes of death in children younger than 5 years in China in 2008. Lancet. 2010;376(9735):89; author reply -90. Epub 2010/07/14. 10.1016/S0140-6736(10)61073-2 20621232

[19] Wang Y, Li X, Zhou M, Luo S, Liang J, Liddell CA, et al Under-5 mortality in 2851 Chinese counties, 1996–2012: a subnational assessment of achieving MDG 4 goals in China. Lancet. 2016;387(10015):273–83. Epub 2015/10/30. 10.1016/S0140-6736(15)00554-1 26510780 PMC5703217

[20] Wen ZQ, Wang H, Yang XR. Design of high-available integration platform for hospital information system. Computer and modernization. 2009;6(6):141.

[21] Human Development Index, 2010. 2013 China National Human Development Report. United Nations Development Programme China. Beijing 2013. p. 105.

[22] Organisation for Economic Co-operation Development Staff. Health at a Glance. The Organisation for Economic Co-operation and Development. Paris; 2005.

[23] Garner JS, Jarvis WR, Emori TG, Horan TC, Hughes JM. CDC definitions for nosocomial infections, 1988. American journal of infection control. 1988;16(3):128–40. Epub 1988/06/01. 2841893 10.1016/0196-6553(88)90053-3

[24] Jing HZ, Huang DM, Guan XJ. Practice of Neonatology. 3 ed Beijing: People's Health Publishing House; 2002.

[25] Caserta MT. Neonatal pneumonia: Merck Publishing; 2015 [cited 2015 October 2015]. http://www.msdmanuals.com/professional/pediatrics/infections-in-neonates/neonatal-pneumonia.

[26] Caserta MT. Neonatal sepsis: Merck Publishing; 2015 [cited 2015 October 2015]. http://www.msdmanuals.com/professional/pediatrics/infections-in-neonates/neonatal-pneumonia.

[27] Kendig JW, Nawab U. Respiratory Distress Syndrome in Neonates: Merck Publishing; 2015 [cited 2015 January 2015]. http://www.msdmanuals.com/professional/pediatrics/perinatal-problems/respiratory-distress-syndrome-in-neonates.

[28] National Bureau of Statistics of China. China population and employment statistics yearbook 2013 China Statistics Press; 2014.

[29] Xi-rong G, Hui Y, Mei H, Xinhui L, Yunqin W, Yimin Z, et al Successful treatment and follow-up of one 500 gram extremely low birth weight infant. Chinese Journal of Perinatal Medicine. 2009;(5):355–8.

[30] 30th anniversary of Neonatal Intensive Care Unit at Children's Hospital of Zhejiang University and celebration by dozens of internationally renowned experts in Hangzhou 2015 [cited 2015 2015-10-16]. http://www.zjuch.cn/Html/News/Articles/4840.html.

[31] Zhang Y, Coordination Group for National Survey of Development in Pediatric and Neonatal Intensive Care Units. Development of pediatric and neonatal intensive care units: results of a national survey (2000–2009). Chinese medical journal. 2011;49(9):669–74. Epub 2011/12/20. 22176901

[32] Lin R, Zhang CM, Tan LH, Shi LP, Xiong QX, Zhang EW, et al Emergency use of extracorporeal membrane oxygenation in pediatric critically ill patients. Chinese Journal of Pediatrics. 2012;50(9):649–52. Epub 2012/11/20. 23158812

[33] Xu T, Wang HS, Ye HM, Yu RJ, Huang XH, Wang DH, et al Impact of a nationwide training program for neonatal resuscitation in China. Chinese medical journal. 2012;125(8):1448–56. Epub 2012/05/23. 22613652

[34] The International Training Program in Neonatal-Perinatal Medicine in Shanghai 2004. http://www.canadianneonatalnetwork.org/portal/CNNHome/TrainingProgram.aspx.

[35] Singhal N, Lockyer J, Fidler H, Aziz K, McMillan D, Qiu X, et al Acute Care of At-Risk Newborns (ACoRN): quantitative and qualitative educational evaluation of the program in a region of China. BMC medical education. 2012;12:44 Epub 2012/06/22. 10.1186/1472-6920-12-44 22716920 PMC3437201

[36] Mukhopadhyay K, Louis D, Mahajan R, Kumar P. Predictors of mortality and major morbidities in extremely low birth weight neonates. Indian pediatrics. 2013;50(12):1119–23. Epub 2013/09/04. 23999672 10.1007/s13312-013-0305-8

[37] Tagare A, Chaudhari S, Kadam S, Vaidya U, Pandit A, Sayyad MG. Mortality and morbidity in extremely low birth weight (ELBW) infants in a neonatal intensive care unit. Indian journal of pediatrics. 2013;80(1):16–20. Epub 2012/11/15. 10.1007/s12098-012-0818-5 23150228

[38] First successfully treatment of an extremely preterm with 24 weeks of gestional age and a birth weight of 500 grams in China by Shengzhen Maternity and Child Healthcare Hospital 2015 [cited 2015 November 12 2015]. http://www.szmch.net.cn/Search/news/371.html.

[39] Meadow W, Lee G, Lin K, Lantos J. Changes in mortality for extremely low birth weight infants in the 1990s: implications for treatment decisions and resource use. Pediatrics. 2004;113(5):1223–9. Epub 2004/05/04. 15121933 10.1542/peds.113.5.1223

[40] Itabashi K, Horiuchi T, Kusuda S, Kabe K, Itani Y, Nakamura T, et al Mortality rates for extremely low birth weight infants born in Japan in 2005. Pediatrics. 2009;123(2):445–50. Epub 2009/01/28. 10.1542/peds.2008-0763 19171608

[41] Wang YP, Miao L, Dai L, Zhou GX, He CH, Li XH, et al Mortality rate for children under 5 years of age in China from 1996 to 2006. Public health. 2011;125(5):301–7. Epub 2011/04/29. 10.1016/j.puhe.2011.01.003 21524772

[42] Smith LK, Draper ES, Field D. Long-term outcome for the tiniest or most immature babies: survival rates. Seminars in fetal & neonatal medicine. 2014;19(2):72–7. Epub 2013/12/03. 24289904 10.1016/j.siny.2013.11.002

[43] Fanaroff AA, Stoll BJ, Wright LL, Carlo WA, Ehrenkranz RA, Stark AR, et al Trends in neonatal morbidity and mortality for very low birthweight infants. American journal of obstetrics and gynecology. 2007;196(2):147.e1–8. Epub 2007/02/20. 17306659 10.1016/j.ajog.2006.09.014

[44] Jones HP, Karuri S, Cronin CM, Ohlsson A, Peliowski A, Synnes A, et al Actuarial survival of a large Canadian cohort of preterm infants. BMC pediatrics. 2005;5:40 Epub 2005/11/11. 10.1186/1471-2431-5-40 16280080 PMC1315360

[45] Meng Q, Xu L, Zhang Y, Qian J, Cai M, Xin Y, et al Trends in access to health services and financial protection in China between 2003 and 2011: a cross-sectional study. The Lancet. 379(9818):805–14. 10.1016/S0140-6736(12)60278-5 22386034

[46] Xu W, Zhang SC. Chinese pediatricians face a crisis: should they stay or leave? Pediatrics. 2014;134(6):1045–7. Epub 2014/11/12. 10.1542/peds.2014-1377 25384495

[47] Hu KJ, Sun ZZ, Rui YJ, Mi JY, Ren MX. Shortage of paediatricians in China. Lancet. 2014;383(9921):954 Epub 2014/03/19. 10.1016/S0140-6736(14)60482-7 24629297

[48] Xiong J, Hipgrave D, Myklebust K, Guo S, Scherpbier RW, Tong X, et al Child health security in China: a survey of child health insurance coverage in diverse areas of the country. Social science & medicine (1982). 2013;97:15–9. Epub 2013/10/29. 24161084 10.1016/j.socscimed.2013.08.006 PMC4181356

[49] Angus DC, Linde-Zwirble WT, Clermont G, Griffin MF, Clark RH. Epidemiology of neonatal respiratory failure in the United States: projections from California and New York. American journal of respiratory and critical care medicine. 2001;164(7):1154–60. Epub 2001/10/24. 10.1164/ajrccm.164.7.2012126 11673202

[50] World Health Organization. Every newborn: an action plan to end preventable deaths. 2014.

