# Supplementary material for: Advanced Neonatal Medicine in China: A National Baseline Database
Source: PLoS One. 2017 Jan 18;12(1):e0169970. doi: 10.1371/journal.pone.0169970 (PMC5242436; doi:10.1371/journal.pone.0169970)
Supplement: S1 Table — (DOCX) [file pone.0169970.s001.docx]

# Supporting Information

**S1 Table: List of participating hospitals**

| **Hospital number** | **Regions** | **Provinces** | **Cities** | **Hospital names** | **Hospital types** |
| --- | --- | --- | --- | --- | --- |
| 1 | North China | Beijing | Beijing | Children's hospital Affiliated to Capital Institute of Pediatrics | CH |
| 2 | North China | Beijing | Beijing | Affiliated Children's Hospital of Capital Institute of Pediatrics | CH |
| 3 | North China | Beijing | Beijing | Peking University First Hospital | GH |
| 4 | North China | Beijing | Beijing | Beijing Maternity Hospital of Capital Medical University | MCH |
| 5 | North China | Beijing | Beijing | Peking Union Medical College Hospital | GH |
| 6 | North China | Tianjin | Tianjin | Tianjin Center Hospital of Obstetrics and Gynecology | MCH |
| 7 | North China | Tianjin | Tianjin | Tianjin Children's Hospital | CH |
| 8 | North China | Hebei | Shijiazhuang | Children's Hospital of Hebei Province | CH |
| 9 | North China | Hebei | Shijiazhuang | Hebei Provincial People's Hospital | GH |
| 10 | North China | Inner Mongolia | Hohhot | Affiliated Hospital of Inner Mongolia Medical College | GH |
| 11 | Northeast China | Liaoning | Shenyang | Shengjing Hospital of China Medical University | GH |
| 12 | Northeast China | Jilin | Changchun | First Hospital of Jilin University | GH |
| 13 | Northeast China | Jilin | Changchun | Changchun Children's Hospital | CH |
| 14 | Northeast China | Heilongjiang | Harbin | Harbin Children's Hospital | CH |
| 15 | Northeast China | Heilongjiang | Harbin | First Affiliated Hospital of Harbin Medical University | GH |
| 16 | East China | Shanghai | Shanghai | Children's Hospital of Fudan University | CH |
| 17 | East China | Shanghai | Shanghai | Shanghai Children's Hospital | CH |
| 18 | East China | Shanghai | Shanghai | Shanghai Children's Medical Center | CH |
| 19 | East China | Jiangsu | Nanjing | Nanjing Children's Hospital | CH |
| 20 | East China | Jiangsu | Suzhou | Children's Hospital of Soochow University | CH |
| 21 | East China | Zhejiang | Hangzhou | Children's Hospital of Zhejiang University School of Medicine | CH |
| 22 | East China | Zhejiang | Wenzhou | Second Affiliated Hospital of Wenzhou Medical College | GH |
| 23 | East China | Anhui | Hefei | Anhui Provincial Children's Hospital | CH |
| 24 | East China | Anhui | Hefei | First Affiliated Hospital of Anhui Medical University | GH |
| 25 | East China | Fujian | Fuzhou | Maternal and Child Health Care Hospital of Fujian Province | MCH |
| 26 | East China | Jiangxi | Nanchang | Jiangxi Children's Hospital | CH |
| 27 | East China | Jiangxi | Nanchang | First Affiliated Hospital of Nanchang University | GH |
| 28 | East China | Shandong | Jinan | Second Hospital of Shandong University | GH |
| 29 | East China | Shandong | Jinan | Qilu Hospital of Shandong University | GH |
| 30 | East China | Shandong | Jinan | Shandong Provincial Hospital | GH |
| 31 | South Central China | Henan | Zhengzhou | Zhengzhou Children's Hospital | CH |
| 32 | South Central China | Henan | Zhengzhou | Third Affiliated Hospital of Zhengzhou University | MCH |
| 33 | South Central China | Hubei | Wuhan | Tongji Hospital of Huazhong University of Science and Technology | GH |
| 34 | South Central China | Hubei | Wuhan | Women and Children's Hospital of Hubei Province | MCH |
| 35 | South Central China | Hubei | Wuhan | Wuhan Children's Hospital | CH |
| 36 | South Central China | Hubei | Wuhan | Union Hospital of Huazhong University of Science and Technology | GH |
| 37 | South Central China | Hunan | Changsha | Third Xiangya Hospital of Central South University | GH |
| 38 | South Central China | Hunan | Changsha | Hunan Children's Hospital | CH |
| 39 | South Central China | Hunan | Changsha | Xiangya Hospital of Central South University | GH |
| 40 | South Central China | Guangdong | Canton | Guangzhou Women and Children's Medical Center | MCH |
| 41 | South Central China | Guangdong | Shenzhen | Shenzhen Maternity and Child Healthcare Hospital | MCH |
| 42 | South Central China | Guangdong | Canton | Guangdong Women and Children's Hospital | MCH |
| 43 | South Central China | Guangdong | Canton | Guangdong Provincial People's Hospital | GH |
| 44 | South Central China | Guangdong | Canton | Nanfang Hospital of Southern Medical University | GH |
| 45 | South Central China | Guangdong | Canton | First Affiliated Hospital of Sun Yat-sen University | GH |
| 46 | South Central China | Guangxi | Nanning | Maternal and Child Health Care Hospital of Guangxi Province | MCH |
| 47 | South Central China | Guangxi | Nanning | First Affiliated Hospital of Guangxi Medical University | GH |
| 48 | Southwest China | Chongqing | Chongqin | Children's Hospital of Chongqing Medical University | CH |
| 49 | Southwest China | Sichuan | Chengdu | West China Second Hospital of Sichuan University | MCH |
| 50 | Southwest China | Guizhou | Guiyang | Guiyang City Children's Hospital | CH |
| 51 | Southwest China | Yunnan | Kunming | Kunming Children's Hospital | CH |
| 52 | Southwest China | Yunnan | Kunming | First People's Hospital of Yunnan Province | GH |
| 53 | Southwest China | Yunnan | Kunming | First Affiliated Hospital of Kunming Medical College | GH |
| 54 | Northwest China | Shanxi | Xi'an | Shanxi Provincial Maternal and Child Health Hospital | MCH |
| 55 | Northwest China | Shanxi | Xi'an | First Affiliated Hospital of Xi'an Jiaotong University | GH |
| 56 | Northwest China | Gansu | Lanzhou | Gansu Provincial People's Hospital | GH |
| 57 | Northwest China | Gansu | Lanzhou | Second Hospital of Lanzhou University | GH |
| 58 | Northwest China | Qinghai | Xining | Women and Children's Hospital of Qinghai Province | MCH |
| 59 | Northwest China | Ningxia | Yinchuan | General Hospital of Ningxia Medical University | GH |
| 60 | Northwest China | Xinjiang | Urumqi | Xinjiang Uygur Autonomous Region People's Hospital | GH |
| 61 | Northwest China | Xinjiang | Urumqi | First Affiliated Hospital of Xinjiang Medical University | GH |

CH, child hospital; GH, general hospital; MCH, maternal and child hospital.
